# An Automated Canine Line-Up for Detection Dog Research

**DOI:** 10.3389/fvets.2021.775381

**Published:** 2021-12-20

**Authors:** Edgar O. Aviles-Rosa, Shawna F. Gallegos, Paola A. Prada-Tiedemann, Nathaniel J. Hall

**Affiliations:** ^1^Canine Olfaction Research and Education Laboratory, Department of Animal and Food Sciences, Texas Tech University, Lubbock, TX, United States; ^2^Forensic Analytical Chemistry and Odor Profiling Laboratory, Department of Environmental Toxicology, Texas Tech University, Lubbock, TX, United States

**Keywords:** olfactometer, detection dog, automated line-up, research equipment, olfaction

## Abstract

Currently, there is a need to develop technology that facilitates and improves detection dog research. The aim of this research was to develop an automated computer-driven olfactory line-up task. The apparatus consisted of three olfactometers. Each olfactometer was equipped with flow meters to regulate air flow and dilution and six solenoid valves connected to odor jars. Each olfactometer generated an odor which was carried to an odor port where the dogs sample it. The olfactometer's valves were activated by a microcontroller, and a Python program was built to control each olfactometer and randomize and balance the odor presentation. Dogs (*N* = 12) received one or two 40-trial training sessions in a day where they progressed through a series of training phases where they learned to detect and alert to double-base smokeless powder (SP). An “alert” consisted of a 4-s nose hold. This was measured by infrared sensors in the ports. For each trial, the apparatus recorded dogs' search latency, sniff time, port entries, and response. All this information was automatically recorded in a csv file. A photoionization detector (PID) and solid-phase microextraction followed by gas chromatography-mass spectrometry (SPME-GC/MS) were used to evaluate the odor dynamics and to instrumentally verify odor presence and clearance. A control test was conducted at the end of the training to ensure dogs were alerting exclusively to the odorant. All 12 dogs readily learned to operate the apparatus within 23 days, and all exceeded 85% accuracy. Control tests indicated dogs were leveraging only olfactory cues and not unintentional cues such as auditory cues from the apparatus. Analytical data showed that odor was detected in the port immediately after the activation of a valve and that odor clearance occurred immediately after the valve was closed. The apparatus developed was easy to operate by the dogs and allowed substantial data collection using double-blind testing procedures in a very short period at an affordable cost point for research equipment (~$5,000 USD). The apparatus may prove to be a useful research tool to provide optimal odor stimuli control, ensure double-blind conditions, reduce labor, and significantly increase the amount of data collected.

## Introduction

Detection dogs' broad and important use for homeland security and military applications highlights the need for research and development in detection dog proficiency assessments and training. Although technological advances have been made for equipment a dog may use in the field such as hearing (e.g., EAR PRO^TM^; https://www.rexspecs.com/) and eye protection (e.g., Rex Specs^TM^; https://www.rexspecs.com/), little development has been done to advance technology that improves dog training proficiency and assessment or the development of research tools to advance this field [see notable exceptions: (1, 2)].

Detection dogs are frequently tested in “line-up” procedures or “odor recognition tests” in which a series of items (suitcases, paint cans, etc.) are placed in a line or circle and the dog is asked to search them. Such procedures are frequently used for research as well (3–7). Line-up procedures are commonly used in training and research because they are inexpensive, easy to setup, and can be adapted to different settings. Nevertheless, line-up paradigms have their limitations particularly for research purposes. The main limitation of these procedures is that conducting double-blind testing, where both the dog and the handler are blind to the presence of an odor, is manually time-consuming and requires significant labor. In a line-up, double-blind conditions are ensured by having multiple experimenters in addition to the dog handler. Ensuring double-blind conditions is critical, particularly for research purposes, as a handler's knowledge or beliefs of the presence of a target can influence dogs' behavior (4, 8). In addition, when using a line-up, additional efforts are necessary to control for a variety of other potential cues dogs may leverage (scent marks on the samples from previous dogs, memorizing odor order on repeated trials, using unintentional auditory cues, etc.). Furthermore, each trial in a line-up procedure requires substantial effort to prepare odorant placement, limiting the data collection capacity of the experimenter. Some of the limitations of a line-up paradigm can be overcome by the development of automated systems that can present samples to the dog, detect dogs' responses, and collect the data automatically [e.g., (2)]. The development of an automated system with these capabilities will ensure double-blind conditions, maximize data collection, and reduce labor. This will result in more accurate and reliable data in detection dog research.

Different attempts have been made to develop automated systems in the past. Mancini et al. (9) used pressure sensitivity pads to automatically record cancer-screening dogs' responses to different samples. This system automated the recording of a dog's responses, but the sample presentation and the delivery of the reward continued to be manual. Recently, Edwards (1) designed a scent wheel that automated data collection and the delivering of the reinforcer. Edwards trained dogs to rotate the scent wheel by activating an omnidirectional switch until they found a target odor. The dogs were further trained to alert to a target odor by holding their nose in the sample port for at least 1 s. Infrared beam sensors were used to measure the nose hold duration, and a computer program recorded dog responses. A correct response resulted in the activation of a feeder to deliver a food reward. This system moved the field forward by providing an automated and controlled “scent wheel,” but it still has some applicable limitations. For instance, training dogs to operate the wheel can be challenging, and the paradigm does not resemble detection dog training. Furthermore, odor samples are still needed to be placed manually in the wheel, and the system did not have the odor control that is available with other automated systems such as olfactometers. More recently, Jendrny et al. (2) used a novel automated system to train COVID-19 detection dogs. This system shows promise; however, details of the apparatus and operation were not fully detailed for replication.

An olfactometer is an instrument that uses odor-free air to carry an odorant and present it to a participant for evaluation (10, 11). In general, olfactometers use filtered or compressed air to carry the headspace of an odor jar into a sampling port where the subject can smell it. Using computer-controlled valves, olfactometers allow the experimenter to control the odor concentration and the duration of odor exposure, resulting in optimal odorant stimulus control during testing. Because of this, olfactometers have been commonly leveraged for olfactory threshold and discrimination studies in rodents (12), humans (13), and dogs (5, 14–20). However, these systems typically have a single port and do not frequently represent the more frequently used “line-up” that deployed detection dogs are frequently trained on.

Thus, to further advance and improve detection dog research and bridge gaps between research methodologies and training used for operational detection dogs, there remains the need to develop a behavioral training and testing apparatus that combines the benefits of odorant stimulus control of an olfactometer but does so in a more realistic search setting such as a “line-up” in which detection dogs are routinely evaluated. The aim of this paper is to develop an automated computer-driven olfactory line-up task for dogs that resembles a search scenario, leverages olfactometer odor stimulus control, and objectively and automatically scores dog responses. Our objective is to provide details about the apparatus schematics and training procedure such that other researchers can use, improve, and further advance the detection dog research field.

## Materials and Methods

### Apparatus

#### Olfactometer Line-Up Design

**Figure 1:**
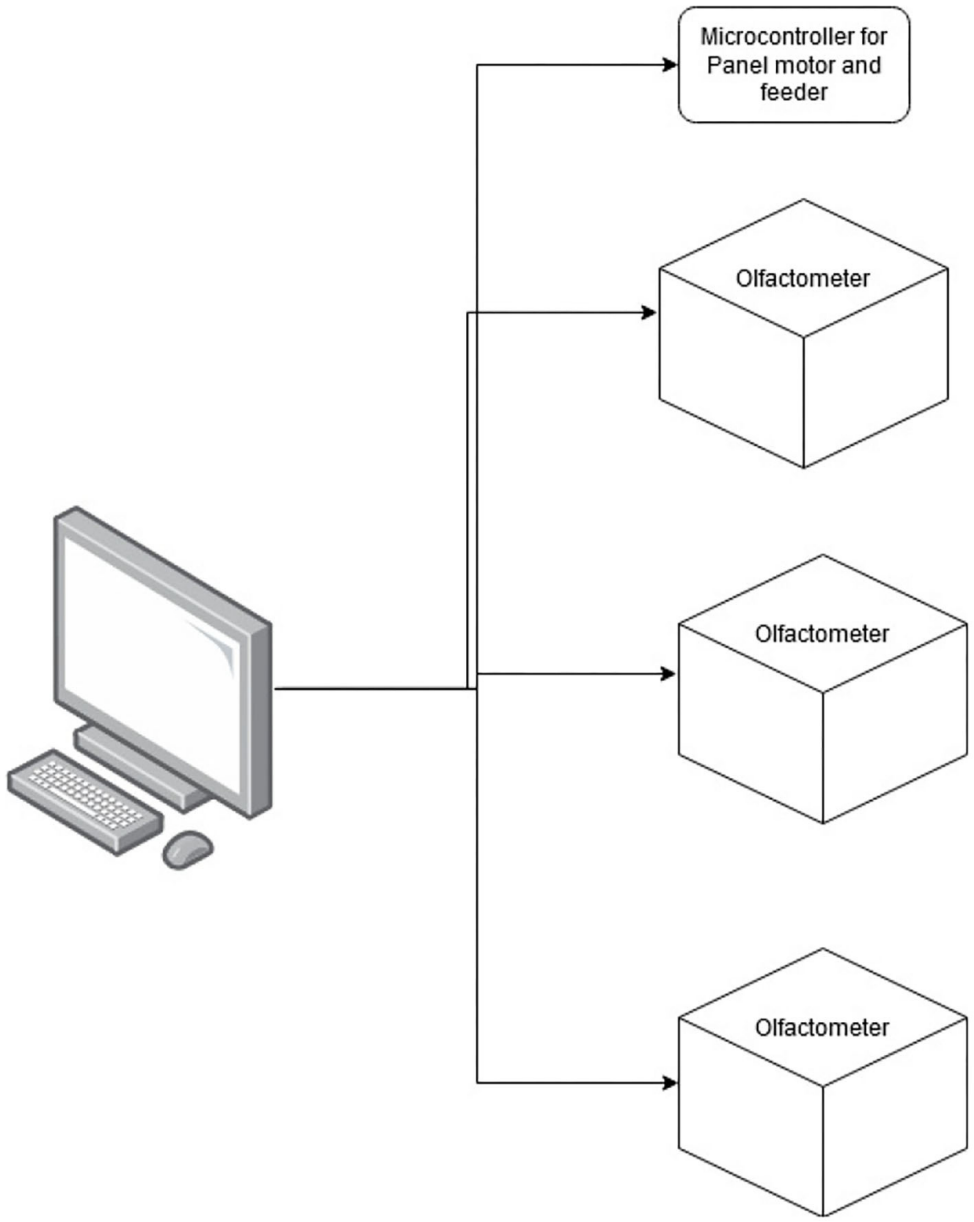
Outline of the line-up design. The figure illustrates how the three independent olfactometers and the microcontroller for the panel and feeder were operated by a central computer.

**Figure 2:**
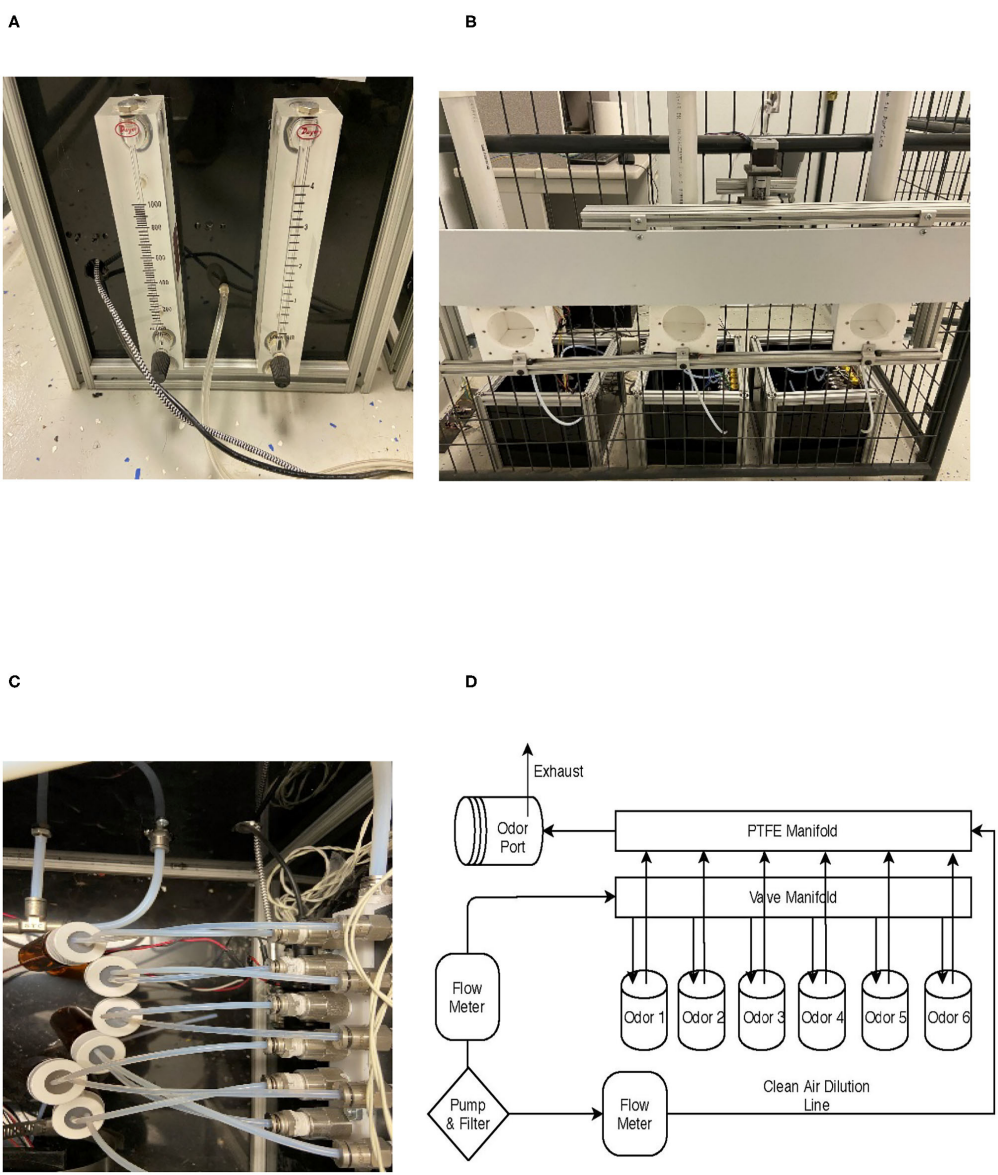
Olfactometer design. **(A)** Common air source and rotameter air flow control. **(B)** Three ports and panel cover that moves up and down to cover the ports. **(C)** Jars connected to manifolds. **(D)** Overall air flow design for a single olfactometer.

To produce a three-alternative line-up for dogs to search, three separate olfactometers, each one with six odor channels, and a panel were produced. Each olfactometer was controlled by its own microcontroller, and each olfactometer independently controlled the odor presented to one of the three sampling ports. The olfactometers were controlled *via* a central computer (Figure 1) that interfaced with a microcontroller (Arduino Nano BLE Sense^TM^ board) that controlled the olfactometer's valves. The central computer also interfaced with a fourth microcontroller that was responsible for driving up and down a motor (100-mm Linear Rail Guide with NEMA17 stepper motor) that held a panel covering the olfactometer ports (Figure 2). Additionally, this microcontroller interfaced with an automated feeder (PetTutor^TM^) *via* Bluetooth, which delivered the food treats. A list of the parts, manufacturers, order information, and costs are included in the Supplementary Material.

**Figure 3:**
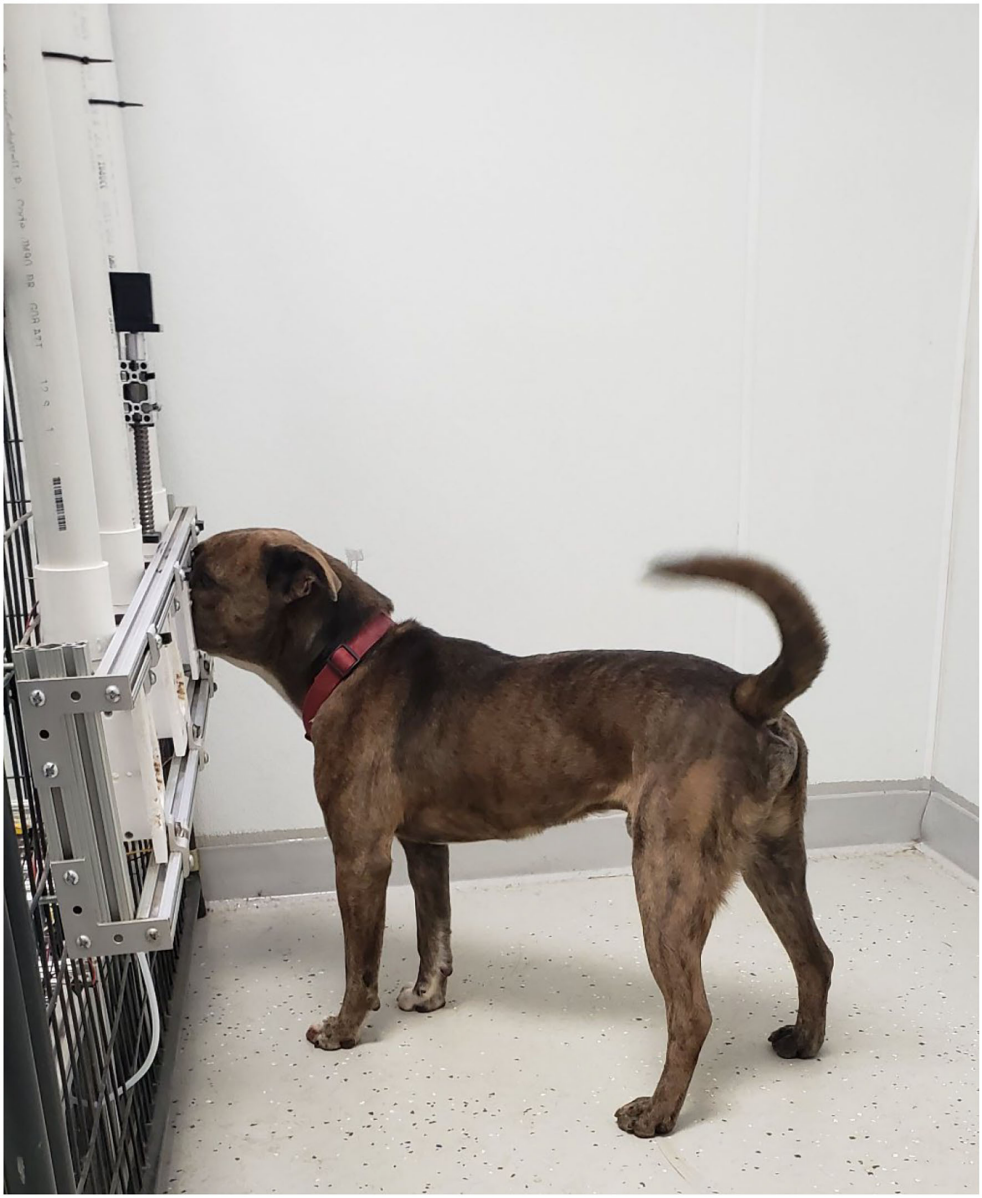
Buster Alerting to the port containing the target odor. Infrared (IR) sensors located at the front of the port measure the nose hold duration, providing an objective recording of the dogs' alert.

Three polytetrafluoroethylene (PTFE; Teflon^TM^) sampling ports were mounted to an aluminum T-slot frame and spaced 35.5 cm apart and were 58.4 cm from the ground (Supplementary Table: ref 16 and 21). The PTFE ports were mounted to the side of a panel (Figure 2B). This panel was moved up and down by a motor to allow or prevent dogs' access to the ports at the beginning or end of each trial, respectively. At the bottom of each odor port was a 1/8″ NPT to tube fitting by which the odorant was introduced to the sampling port (Supplementary Table: ref 13). At the top of each port was a ¾″ NPT fitting (Supplementary Table: ref 25) connected to a 3.8-cm PVC fitting. Inside the PVC fitting was a 40 × 40-mm fan (7 CFM), which served to exhaust the odorant (Supplementary Table: ref 23) at the end of each trial (Figure 2B). The fan would blow the odorant along a PVC pipe to exhaust it out of the room (Supplementary Table: ref 25). Infrared beam sensors (Supplementary Table: ref 24) were mounted to the T-slot channel on the outside entrance of each odor port to measure canine nose port entries and to record the duration of the nose hold to evaluate dogs' response (Figure 3).

Odor was generated by each olfactometer independently. Each olfactometer was fed from a common air supply generated by a high-flow air pump (Supplementary Table: ref 26) and cleaned using a charcoal filter (Supplementary Table: ref 27). The common airline connected to the back of the olfactometers (Figure 2A), which was regulated by two rotameters (Dwyer VFB^TM^; Supplementary Table: refs 3 and 4). One rotameter regulated a clean airline (Figure 2D) and ranged from 1 to 4 l/min (LPM), and the other rotameter regulated the odor line between 0.1 and 1 LPM. The clean airline was directly connected to a final PTFE manifold (white manifold Figure 2C; Supplementary Table: ref 10), which flowed unrestricted to the odor port. This provided continuous airflow for clearing the odor from the port between trials and dilute the odorant. The odor line was connected to a manifold bank with six 12-V solenoid valves (Supplementary Table: refs 1–2). Stainless steel push-to-connect fittings connected a 0.16 ID × 0.32 OD PTFE tubing (Supplementary Table: ref 8) from the manifold to a borosilicate glass jar containing the odorant. The lid of the glass jar was a lid designed for VOC sampling with a PTFE and silicone septa lid. A small hole was pierced with stainless steel tubing, which allowed the PTFE tubing to be directly inserted into the lid (Figure 2C). A second exit line was also inserted into the jar, connecting a path for the headspace to exit when air was introduced. The exit airline was connected to a stainless steel check valve (Supplementary Table: ref 15), to prevent backward flow, and then to the final PTFE manifold (Supplementary Table: ref 9). In the manifold, the odorant air is mixed with the continuous clean line and exited at the odor port for the dog to sample (Figure 2D).

### Electronics Design

A custom-designed printed circuit board (PCB) was made to control each olfactometer, the panel, and the feeder. The PCB integrated an Arduino Nano BLE 33 as a microcontroller; a 12-V power supply; a Darlington transistor array; and breakouts for a motor drive, infrared (IR) beam pairs, solenoids valves, and other sensor peripherals. The.brd file and the.sch file are available in the GitHub link provided in the Supplementary Material section and can be used to order at PCB manufacturers.

### Construction

The olfactometer itself was built as a simple cube with 30.5-cm sides. T-slot metal channel served as the frame with 0.63-cm polypropylene plastic fitted within the T-slot. The construction was custom built and can be customized to fit any desired size and shape.

### Programming

A microcontroller code (Arduino code) was developed with a simple communication interface between the computers to activate olfactometer valves. The code is available in the GitHub link provided in the Supplementary Materials, and the same code can be uploaded to each olfactometer as long as each olfactometer is given a unique name. For the computer, a training program was developed for use with Python 3.x or higher. The Python program is also available *via* the link in the Supplementary Material. The Python program was built to control each olfactometer and randomize and balance the odor presentation in each port. The program was built to randomize the odor presentation but ensures that the odor appeared approximately the same number of times in each port. This program can be modified for different research or training purposes. For instance, experimenters can easily change the algorithm to change the odor presentation rate and reinforcement schedules and to activate multiple valves at a time to create odor mixtures.

#### Data Output

The program outputs a CSV file for each instance of the program. The file is labeled with the entered dog name and a timestamp. The data sheet produces a row for each trial completed, storing information put in by the user and trial specific information. This includes the odors presented in each port, the timestamp of the start of the trial, the latency for the dog to start searching (first nose entry), the number of nose entries to each port, the total sniffing duration to each port, and a list record of each poke and its duration. An example data sheet is provided in the Supplementary Material.

### Subjects

The study was conducted at the Texas Tech University (TTU) Canine Olfaction Research and Education Lab (CORE). For this study, we tested two independent cohorts of six mixed-breed dogs (Table 1). Cohorts were tested 4 months apart from each other. This was due to space limitations in our facility. All dogs had no previous experience or training in scent detection. Participants were selected from local shelters and rescue organizations as a partnership for our train-for-adoption program. Dogs were selected based on their food motivation, size (20–30 kg), age (<10 years), and boldness (e.g., were not afraid and approached the experimenter during the selection trials). Dogs were housed in indoor kennels (2.43 × 1.22 m) in a climate-controlled room with free access to an outdoor kennel (2.43 × 1.22 m). Dogs received 25% of their daily food ration in the morning (~08:00) and the remaining in the afternoon (~16:00). Dogs had free access to water in their kennels and during the training sessions. In addition, dogs received two daily walks and/or play sessions in between training as part of our lab enrichment program. All procedures and animal handling were approved by the TTU Institutional Animal Care and Use committee (protocol # 19093-10).

**Table 1:** Dog information.

**Name**	**Cohort**	**Reproductive status**	**Approximate age (years)**	**Visual breed appearance**	**Average weight (kg)**
Bruce	One	Neutered male	3	Mixed	24.02
Bullseye	Two	Neutered male	2	Mixed	22.50
Buster	Two	Neutered male	3	Mixed	21.50
Charles	Two	Neutered male	1	Husky mix	23.00
Charm	One	Spayed female	1	Lab mix	22.43
Dale	Two	Neutered male	4	Mixed	30.00
Maxine	One	Spayed female	2	German Shepherd mix	24.20
Phantom	One	Neutered male	1	Lab mix	26.28
Pumpkin	One	Neutered male	1	Lab mix	28.16
Raven	One	Spayed female	1	Lab mix	22.76
Sasha	Two	Spayed female	9	Lab mix	22.00
Wishbone	Two	Neutered male	2	Mixed	21.25

### Training

For training, the odor line air flow was set to 1 LPM, and the continuous airline was set to 2 LPM to produce a 33% air dilution of the target odorant. This air dilution was also used for the distractors. Dogs received one or two 40-trial sessions a day based on trainer availability. Dogs were trained up to 5 or 6 days in a week. If dogs were trained twice in a day, each session was at least 2 h apart from each other. During a trial, one sampling port had the target odor, and the other two ports had distractor odors unless it was a blank trial where none of the ports contained the target odor. Dogs progressed through a series of training phases where we trained them to alert to the target odor and ignore the distractors. Training phases were set within the Python program developed for this system and are described in detail below. These training phases show how we conducted our training, but they can be modified based on individual dog performance or trainer experience and expertise.

#### Phase 1

During this phase, we used a biologically interesting odor (food) to promote the search of the apparatus. In this case, the odor of hotdog was used in the initial training phase. The remaining odor channels were filled with distractor (non-target) odors including the following: an empty vial, cotton gauze, latex glove, mineral oil, and limonene (10^−3^ *v*/*v* dilution in mineral oil). These distractors were selected as they are common laboratory ingredients to prepare odorants and include a novel strong odor (limonene). The same distractors were used throughout the different training phases. During training phase 1, the computer interface showed the port that contained the target odor. This was to allow the handler to reinforce correct responses or approximations and facilitate and accelerate training.

During the first training session, dogs were introduced to the room with the equipment off. This was to habituate the dogs to the room. During this habituation period, the handler reinforced every time the dog spontaneously investigated the ports. Once dogs were comfortable in the room and investigating the ports, the handler turned on the air pump to habituate dogs to the sound of the pump. Dogs were usually habituated to the room within 10–20 min. After habituation, the computer program was initiated, and odor trials started. Each trial started by lifting the panel covering the three odor ports and terminated when the dog held its nose in the correct port by the duration criterion set by the handler. At the beginning of the trial, the handler prompted the dog (e.g., tapping the panel with their finger) to investigate all three ports. A nose hold criterion of 0.25 s was initially set, such that initial investigation of the correct port triggers a “beep” from the computer and activates the feeder delivering a food reward. Incorrect responses (i.e., a 0.25-s hold to an incorrect port) were scored as incorrect but did not have any programmed consequences. If a dog failed to trigger the IR sensor at the 0.25-s criterion for the correct port after 20 or more seconds, the handler placed a treat in the correct port, to prompt the dog to enter and hold their nose. This will trigger the IR beam and feeder. After a correct response in each trial, the panel goes down covering the ports, and the exhaust fans were activated for 15 s to clear the odorants from the ports before the initiation of the following trial.

The location of the target odor and distractor odors was randomized by the computer program for each trial. The trial randomization occurs by first determining (1) whether a trial will contain a target odor, (2) which port will contain the target if that trial was programed to contain a target odor (counterbalanced across the three ports), and (3) randomly selecting between the five distractor odors for the non-target olfactometers independently. Thus, each olfactometer selects the distractor independently, and in most trials, the two distractors would be different but could also be the same (if the same odor happens to be selected by the two distractor olfactometers for that trial).

Training with a nose hold criterion of 0.25 s continued until dogs independently searched the line-up at the handler's command (e.g., “search” or “find it”) and activate the IR beams on their own. The nose hold criterion was then increased in 0.5-s steps after every session a dog independently activated the IR beams until reaching a 2-s nose hold criterion. Once reaching the 2-s criterion, accuracy was assessed daily by calculating the number of trials the dogs made a 2-s nose hold only to the port presenting the target odor, and not to other ports. If the dog reached 85% correct responses or higher in a day, the target odor was changed from a biologically interesting odor (hotdog) to a main target odor. For this study, we used double-base smokeless powder (Hodgdon®) as the main target odor. To facilitate odor transition, during the first session with smokeless powder (SP), the nose hold criterion was reduced from 2 to 1 s, and the handler marked (with a clicker or a “yes”) every time the dog poked the correct port. This was done for their first 10 trials of the session, and after, the dog had to alert on its own. If the dog showed problems transitioning to SP after trial 20, the handler marked again the correct port to prevent extinction. Training with SP continued until dogs averaged at least 85% correct responses in two consecutive training sessions. At this point, the nose hold criterion was increased from 2 to 4 s in 0.5-s steps. The step size and return to a previous step size was made based on trainer expertise and dog's performance in a session. Once dogs reached an accuracy of 85% or higher with a 4-s nose hold criterion, dogs advanced to phase 2. If a dog had difficulty or showed a drop in motivation, the dog could return to a previous level of training including re-introducing food odor to re-build motivation. This was done on occasion for some dogs.

#### Phase 2

In phase 2, the computer program was advanced such that testing occurred blinded (the computer did not show the port containing SP) and provided consequences for a false alert or incorrect responses. If a dog made a false alert, the trial would terminate without the food reward. Phase 2 training continued until dog performance was 85% correct or higher in two consecutive sessions. If a dog showed decreased motivation or continued poor performance at this level, the dog could be returned to a previous phase based on trainer expertise.

#### Phase 3

This phase introduced blank trials in which no port contained the target odor. This occurred on 10% of the trials (e.g., 4 out of the 40 trials), and they were randomly distributed within each block of 10 trials. If the dog alerted to a non-target odor, the trial was scored as incorrect and terminated without reward. In addition, during this phase, we also added a time limit of 45 s for a dog to search the ports and make a response. If the dog failed to search all three ports within 45 s, a “timeout” was recorded, and the trial was terminated and scored as incorrect. If a dog searched all three ports and did not alert to any port after 4 s of searching the last port, an “all-clear” response was scored. An all-clear response was recorded as a correct response during blank trials and an incorrect response for trials in which SP was presented. Correct all clears were not reinforced with food. The reinforcer for an all-clear response was to simply advance to the next search, because this is common practice with detection dogs (e.g., if a dog does not find a target in one room, they move onto the next). A dog was considered fully trained if they scored at least 85% correct responses in phase 3 training for two consecutive sessions.

#### Control Testing

To verify dogs were utilizing olfactory cues and not unintentional cues delivered by the olfactometer (e.g., solenoid valve “clicks,” unintentional air flow changes, etc.) a control session of 10 trials was conducted after the dog reached training criterion. The control session was identical to a regular session with the exception that the air flow into the odorant jars was “unplugged” such that odorant air was not introduced to the odor port. Everything else was identical, and programmed consequences remained in effect.

### Odor Delivery and Clearance Validation

A photoionization detector (PID, 200B miniPID, Aurora Scientific®, Canada) was used to validate odor delivery of a tracer odorant. The miniPID was placed in the odor port to validate what the dog was receiving when searching a port during and after a trial. With the miniPID inside the port, we activated the odor valve for 30 s (typical duration of a trial). After the 30 s, the odor line was stopped for 30 s to allow odor clearance and activated again. This cycle was repeated 40 consecutive times. For this test, the olfactometer microcontroller (Arduino) algorithm was modified to run the odor cycle automatically and to send a voltage signal to synchronize valve activation with PID readings. Analog voltage readings from the PID and the microcontroller indicating odor activation were sampled at 30 Hz using a LabJack (U6) DAQ.

The tracer odorant used for this was limonene (CAS:5989-54-8) diluted in mineral oil (10^−1^ *v*/*v*) to facilitate odor detection by the sensor due to the PID's poor sensitivity to SP. The odor line was set at 1 LPM and the continuous line was set at 2 LPM. This produced a 33% air dilution, as in training. The continuous airline was on during the odor clearance period.

### Solid-Phase Microextraction Followed by Gas Chromatography-Mass Spectrometry Sampling of Smokeless Powder

To identify the SP target odorant directly from the olfactometer, diphenylamine was selected as the detectable signature of SP due to is abundant presence in the headspace of directly sampled SP using solid-phase microextraction followed by gas chromatography-mass spectrometry (SPME-GC/MS). Prior to the start of sampling, SP was allowed to equilibrate within an odor vial connected to the olfactometer for at least 1 h. Next, the output line of the olfactometer was connected to a clean glass VOC collection vial (236.5 ml), which was pierced with two needles, the first of which was connected to the olfactometer and the second to vent the air pressure to prevent damage to the collection vial. The olfactometer was activated for 30 s at an airflow ratio of 2:1 (2 l/min of clean air with 1 l/min of odor). To prevent the loss of the collected odor volatiles, the airflow needles were immediately removed, and the odor collection vial immediately sealed with parafilm. A polyacrylate (PA) SPME fiber (Supelco, Sigma Aldrich) was then inserted to the collection vial headspace for an optimized extraction time of 3 h, for a total of six replicates.

To validate odorant clearance in the olfactometer line, blank samplings were conducted to determine if any potential contamination existed between trials. Variations of length of tubing, from 1 ft (short line) to 3 ft (long line), were tested as was the application of heat tape to the shorter line (short line with heat tape) to evaluate if added heat could reduce any potential contaminants present in the system. Six replicates were performed for each variation of the blank sampling trials. In each experiment, 40-ml glass SPME vials with a screw cap and PTFE/silicone septa (Supelco, Sigma Aldrich) were used. For olfactometer testing, 10 g of double-base smokeless powder (H335 rifle powder obtained from Hodgdon Powder Company) was used as the odor sample, and an empty sterile vial was used for the blank odor collection.

A 40-ml odor collection vial was pierced with two needles, the first of which was connected to the olfactometer and the second to vent the air pressure to prevent damage to the collection vial. The sample vial containing the smokeless powder was activated for 30 s at an airflow ratio of 2:1 (2 l/min of air with 1 l/min of odor), and this odor was not collected. Another 30-s interval was allowed to pass to mimic the “clearing” time between canine searches. At the conclusion of the 30-s clearing interval, the sterile empty vial was activated for another 30-s interval. This blank sample was collected in the 40-ml collection vial. The airflow needles were removed, and the odor collection vial immediately sealed with parafilm. The SPME fibers were then inserted for a 3-h extraction period. Upon completion of the 3-h extraction time, the fibers were run with the established gas chromatography–mass spectrometry (GC/MS) method to analyze any potential contaminants that may be carried over from the active odor vial and to confirm that the target odorant, diphenylamine, was not remaining in the olfactometer between active trials.

GC/MS was used as the confirmatory technique for the presence of the target odor volatile in the headspace of all collected samples. An Agilent Technologies GC 7890A with an Agilent Technologies 5975C inert XL MSD with triple-axis detector (Agilent Technologies, Santa Clara, CA) was used to separate and analyze the compounds extracted on the SPME fibers. A Rtx®-5 capillary 30 m × 250 μm × 0.25 μm column (Restek Corporation, Bellefonte, PA, USA) was used. Helium was used as a carrier gas at a flow rate of 1.0 ml/min. The temperature ramp was programmed from 40°C to 280°C beginning with a 1-min hold at 40°C and then increasing the temperature to 200°C at 15°C/min with a 1-min hold at 200°C. The temperature was then increased to 240°C at 15°C/min and held for 6.50 min at that temperature. From 240°C, the temperature was increased at 25°C/min to 270°C. The final temperature of 280°C was reached by ramping the temperature at 5°C/min and holding for 4 min. The injector temperature was set at 280°C in split mode at a split ratio of 5:1.

The total run time for analysis was 29.033 min. Mass spectra were repeatedly scanned from 45 to 550 amu. Target compound was identified using the National Institute of Standards and Technology (NIST) (2017) mass spectral reference library and verified with external standard calibration. The criteria for the compounds identified were those with detected peaks greater than or equal to a match quality of 90% or above.

### Data Analysis

By using IR beam sensors in front of each port, the apparatus had the capability to automatically measure and record the latency to search, the number of times a dog searched a port (port entries), the amount of time the dog sniffed each port (sniff time), a timeout, and dogs' response during each trial. We evaluated dogs' performance progress daily. If a dog received two training sessions in a day, we averaged the performance of both sessions to calculate their daily performance. If a dog received only one session in a day, the performance of that session was used as their daily performance. No null hypothesis testing was conducted, as the aim of this paper is to describe dogs' progression during training. The cumulative sniffing time was calculated by adding the amount of time a dog sniffed each port during a trial. The 4-s nose hold from an alert was not included as part of the sniffing time. In the same way, the cumulative number of port entries was calculated by adding the number of times a dog searched each port during a trial. Latency was defined as the time from the beginning of the trial until the dog initially searched any port. A correct response was noted if a dog alerted to the port containing the target odor or did an all clear during a blank trial in phase 3. A false alert was noted when the dog alerted to a port containing a distractor or to a blank trial. If a dog did not alert to the target odor after sampling all three ports, the response was noted as a miss.

To visualize the signal of the PID, the raw voltage readings were filtered with a low-pass Butterworth filter at a scalar of 0.05 of the Nyquist frequency using the signal package of R (21). To address sensor drift of the voltage readings (observed decreases in baseline values across time) of the PID, we used the mean voltage reading of the 30 s prior to odor activation (between activation times) as an offset value that was added equivalently to both the odor “off” and odor “on” values for that odor cycle. The signal from the olfactometer microcontroller was overlaid to visualize odor activation periods to enhance visualization.

All GC/MS data was analyzed using ChemStation software (Agilent Technologies, Santa Clara, CA). Compounds known to be products of the column or sampling process were not included in the analysis.

All statistics were calculated using SAS 9.4 and R studio statistical software.

## Results

**Figure 4:**
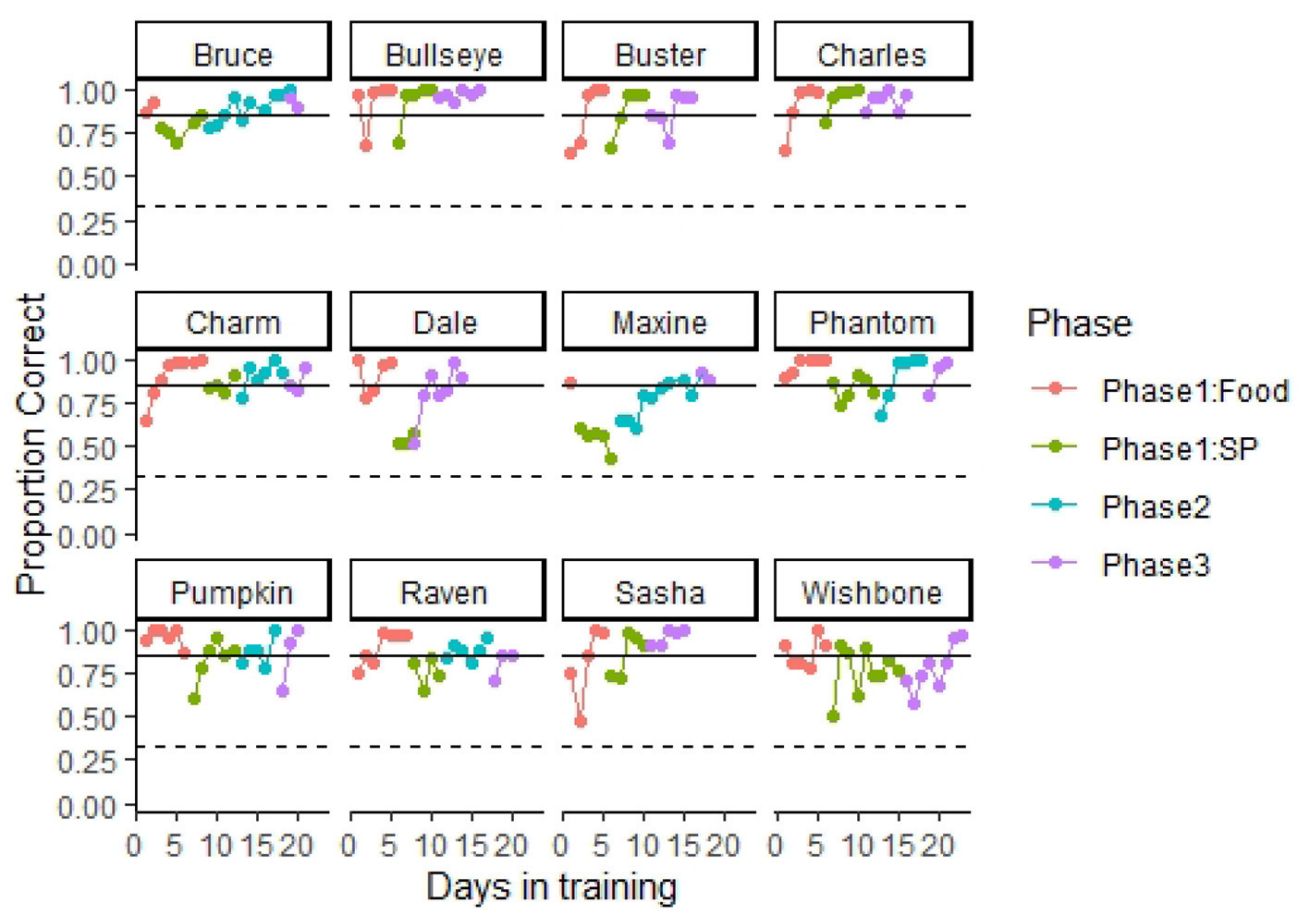
Training progression. The figure shows each dog's proportion of correct responses through the different training phases. A dashed line indicates 0.33 proportion correct responses (performance at chance levels). A solid line indicates 0.85 correct responses (training criterion). All dogs completed training and performed at the olfactometer above training criterion within 23 days in training. Dogs initially were trained using food odor (hotdog) to promote spontaneous interest in search. Next, dogs were transitioned to the target odor smokeless powder (SP). In phase 2, testing was conducted double blind, and trial termination occurred for incorrect responses. In phase 3, blank trials (no target odor present and dogs had to clear all three ports) were introduced at a frequency of 10%.

All 12 dogs presumably naïve to odor detection training learned to operate the equipment and successfully detected SP at accuracy rates exceeding 85% correct responses within 23 days of training. Figure 4 highlights the training progression of each dog through the different training phases. On average, dogs completed the training criterion of phase 1: food in 5.25 ± 0.59 days (7.91 ± 0.80 training sessions). It is important to note that, dogs in our first cohort received some initial training with SP, before starting training with food as we were uncertain and developing the training methods at the time. As we noticed that they were not progressing in training with SP, we decided to implement the initial training with a biologically relevant odor (phase 1: food). Thus, Bruce and Maxine learned the task with the hotdog in only 2 and 1 day, respectively, as they were already familiarized with the equipment. Nevertheless, the rest of the dogs in our first cohort spent similar or even more time in this phase compared to dogs in the second cohort, which only received phase 1: food training. This may suggest that the initial SP exposure had little to no effect accelerating training in most of the dogs. After training with hotdogs, most dogs transitioned to SP without issue. Phantom, Raven, Maxine, and Wishbone each required some return to phase 1: food training during the transition phase. Return to phase 1: food consisted of giving dogs 10 trials with hotdog as the target odor before starting the session with SP to initiate search with the apparatus. On average, within 5.25 ± 0.42 days (8.75 ± 0.70 training sessions), the nose hold alert criterion was increased to 4 s.

Dogs in our fist cohort completed phase 2 training in 7.66 ± 1.08 days (8.83 ± 1.22 training sessions). After phase 1: SP training, we transitioned our second cohort of dogs directly to phase 3. We decided to skip phase 2 with the second cohort based on their performance and trainer experience with the first cohort. After 4.41 ± 0.55 days (4.83 ± 0.73 training sessions) in phase 3, most dogs reached the training criterion of detecting SP at an accuracy >85% with a nose hold of 4 s and a target odor prevalence of 90% of the trials. All dogs met our 85% training criterion during the last two sessions in phase 3 (Table 2), and most reached an accuracy >90% in their final two sessions, with the highest performing dogs reaching 98.75% accuracy (79 correct responses out of 80 trials).

**Table 2:** Mean ± standard error of Dogs' (*N* = 12) average performance during the last two sessions of phase 3.

**Dog**	**Overall accuracy, %**
Bruce	92.50 ± 2.5
Bullseye	98.75 ± 1.25
Buster	95.00 ± 0.00
Charles	92.5 ± 5.00
Charm	87.50 ± 3.82
Dale	93.75 ± 3.75
Maxine	90.00 ± 2.50
Phantom	87.50 ± 7.50
Pumpkin	96.25 ± 3.75
Raven	85.00 ± 0.00
Sasha	98.75 ± 1.25
Wishbone	95.83 ± 1.39

**Figure 5:**
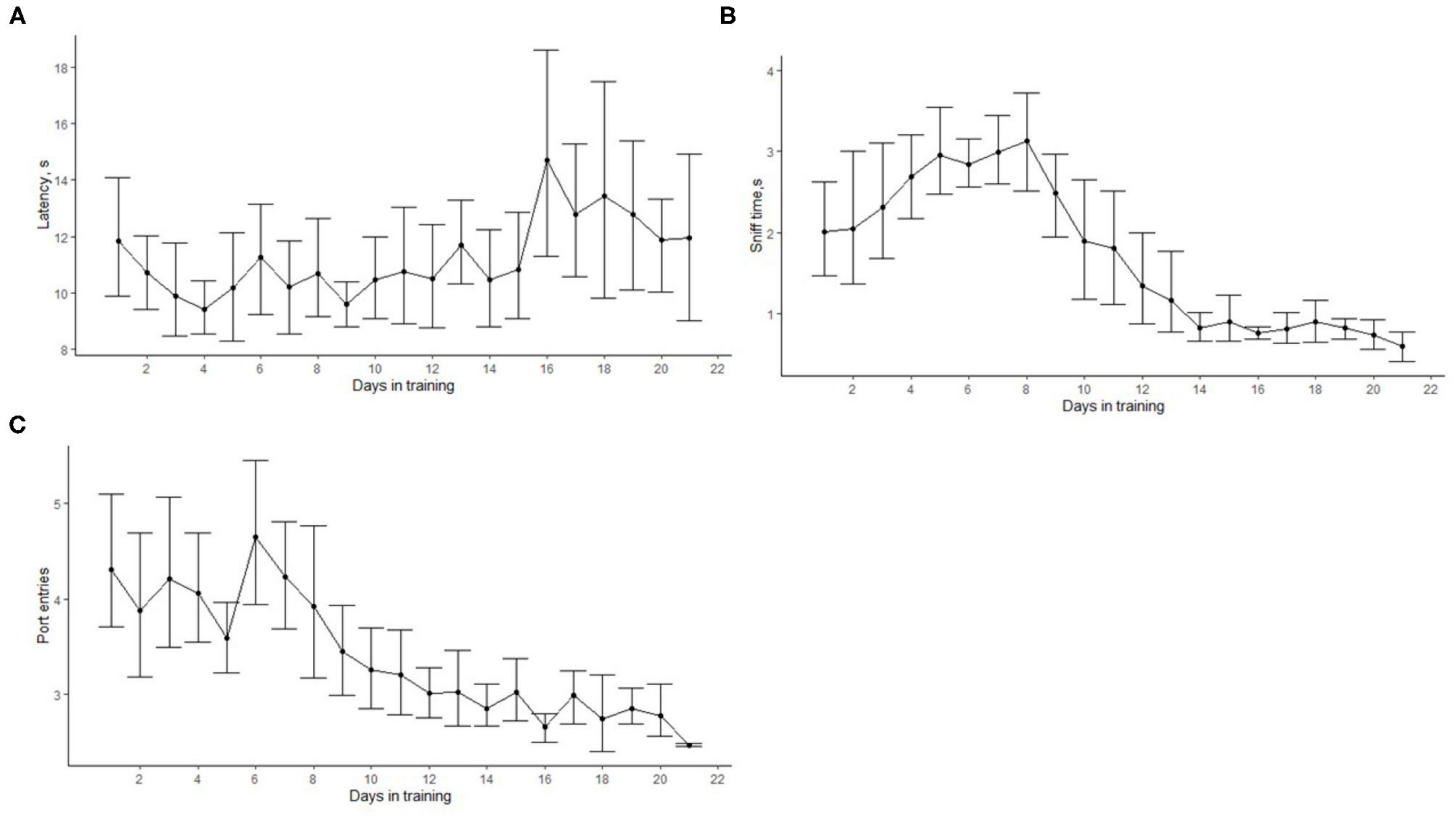
Progression of search-related behaviors in dogs (*N* = 12) within the first 21 training days. The dots show the mean, and error bars show the 95 % confidence interval. The average search latency did not significantly change with time **(A)**. The cumulative sniffing time **(B)** and the cumulative number of port entries within a trial **(C)** decreased with training. This indicates that, as training progressed, dogs were more efficient and were able to detect the target odor more easily.

Figure 5 shows how the latency to search, the number of port entries, and the sniff time changed as training progressed. On average, the latency to search slightly increased after 15 days in training (Figure 5A). This was the time when most dogs were introduced to phase 3. Overall, the average latency to search was 11.05 ± 0.24 s. The number of port entries and the sniffing time reduced with training (Figures 5B,C). At the beginning of the training, the cumulative sniff time (excluding the nose hold required for an alert) was 2.14 ± 0.94 s, and it decreased to <1 s at the end of phase 3. Similarly, the average number of port entries within a trial gradually reduced from more than four at the beginning of training to 2.67 ± 0.05 at the end of phase 3.

**Figure 6:**
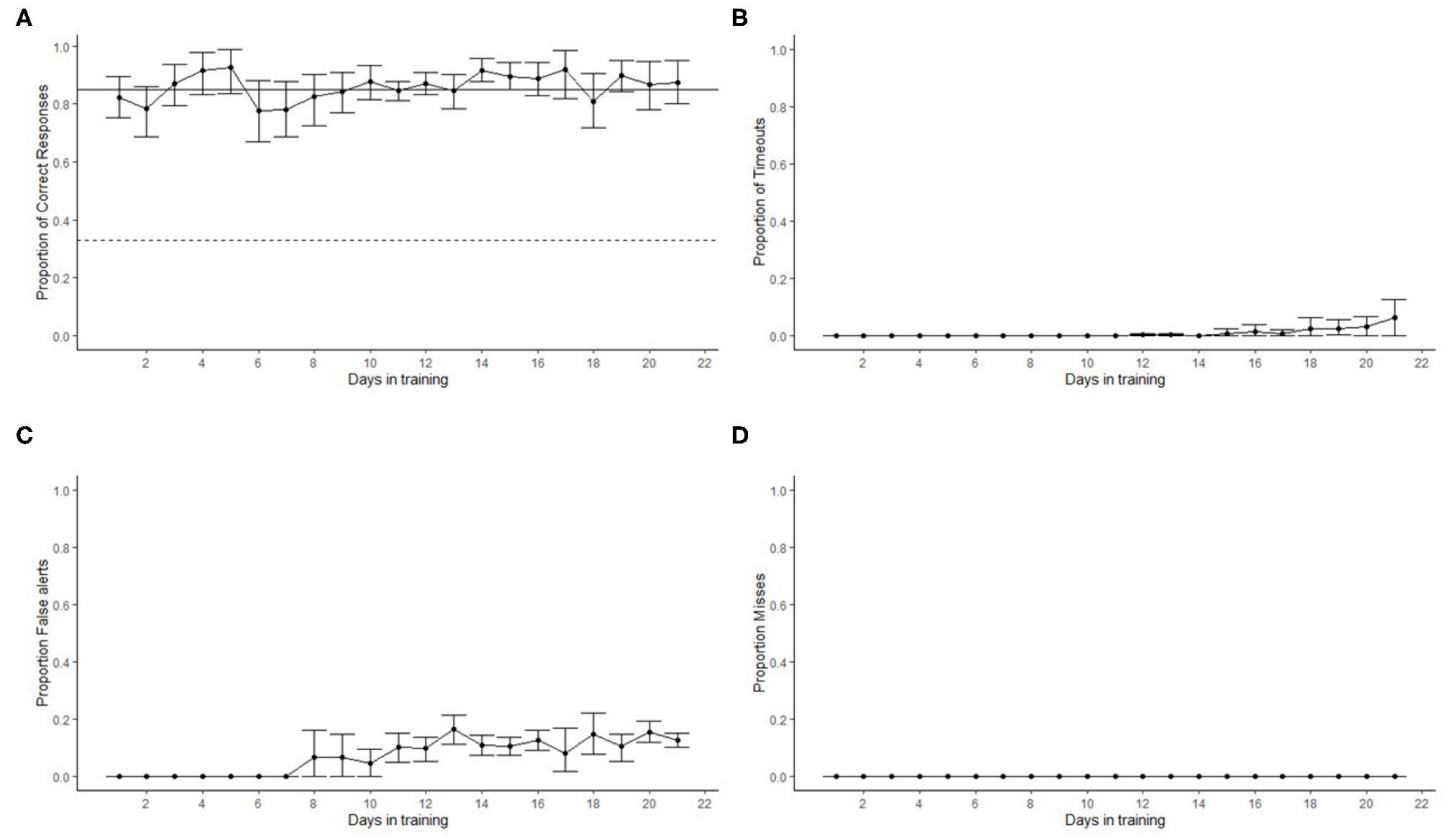
Dogs' (*N* = 12) detection performance in the line-up olfactometer during the first 21 days of training. The dots show the mean and error bars show the 95% confidence interval. **(A)** Dashed line shows chance (0.33) performance and the solid line the training criterion (0.85). Proportion of correct responses was always above criterion within five training sessions. A slight reduction in the proportion of correct responses was observed at day 6. This was the time when most dogs transitioned to smokeless powder (SP). Performance with SP recovered to above criterion levels within four training days. No timeouts were observed until day 15 when most dogs started phase 3, where blank trials were introduced **(B)**. The transition to SP as the target odor from hotdog increased the number of false alerts at day 8, and this continued throughout the remaining of the training **(C)**. No misses were recorded **(D)**.

The overall proportion of correct responses increased above 0.85 by day 3 in training with hotdog as the target odor (Figure 6A). As dogs transitioned to SP, the proportion of correct responses slightly decreased, but overall, the average performance surpassed 0.85 within 4 days of training with SP. All dogs showed a mean performance above the training criterion during phase 3 (days >15), which involved double-blind testing and 10% of the trials as blank trials (dog was required to clear all three ports that did not contain the target). No timeouts were recorded until day 15 (Figure 6B). This was when blank trials (phase 3) were introduced for most of the dogs and because during the initial training a trial did not have a specified termination time. Dogs did not false alert during the training with hotdogs (Figure 6C). Once SP was introduced, the proportion of false alerts increased, but it never averaged more than 20%. Maxine and Dale were the only two dogs that showed a proportion of false alerts >0.20 when introduced to SP training. Because of this, they were moved to training phases 2 and 3 faster than other dogs to penalize (no reinforcement) false alerts and promote correct responses. No misses were recorded during training (Figure 6D). This means that incorrect responses were only due to false alerts or timeouts (failure to sample the target port).

**Figure 7:**
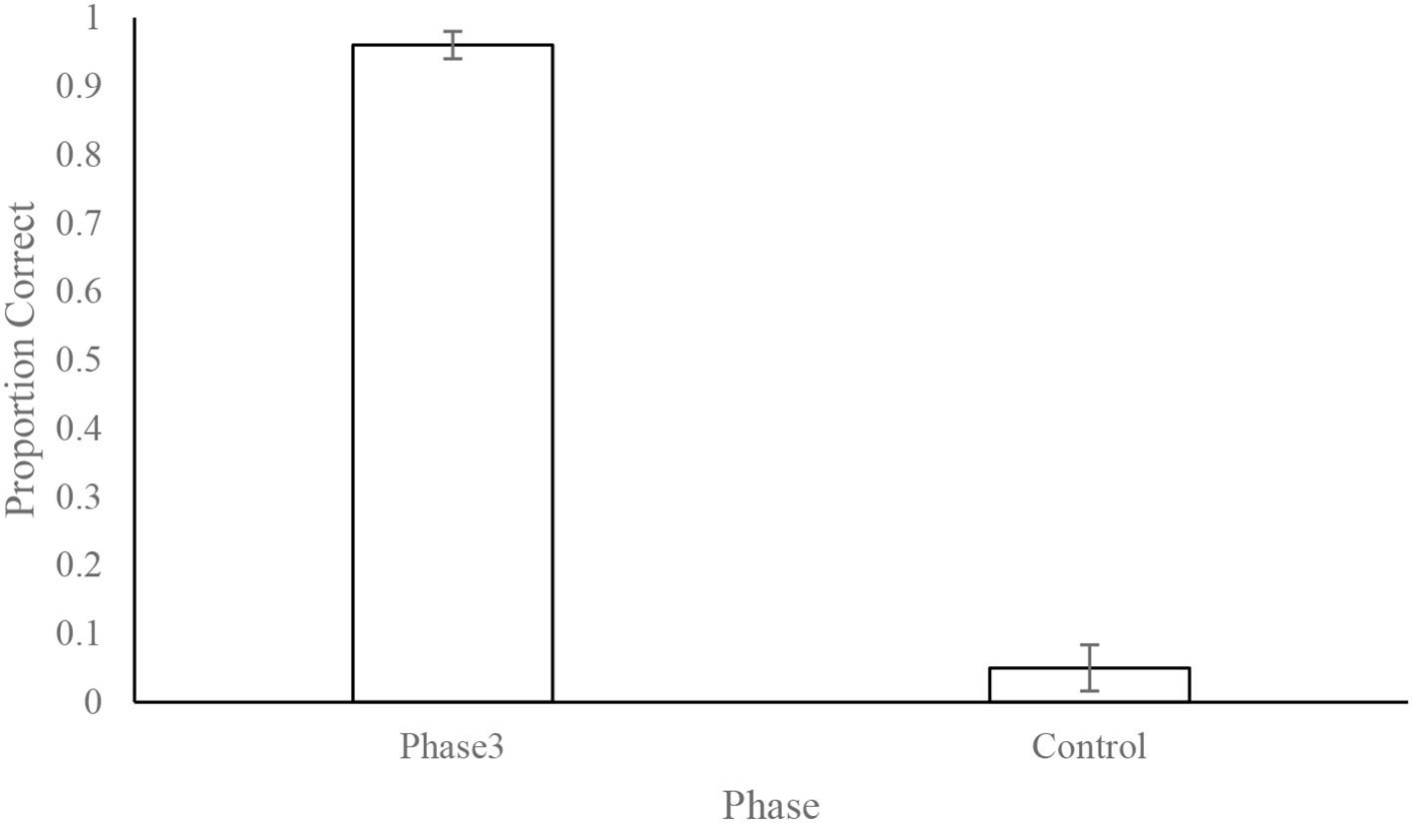
Dogs' (*N* = 12) mean ± standard error of the proportion of correct responses during phase 3 and the control test. The control test consisted of 10 trials where the odor line was disconnected from the olfactometer. This shows that the dogs' performance was mediated by the presence of the odor and not by unintentional cues from the equipment or the handler.

Overall performance on the control session (odorants not connected but valve activated) was poor (5.00% ± 3.37%) and well below that expected by chance (33%), indicating that dogs were indeed following odor cues to identify the correct odor port (Figure 7).

**Figure 8:**
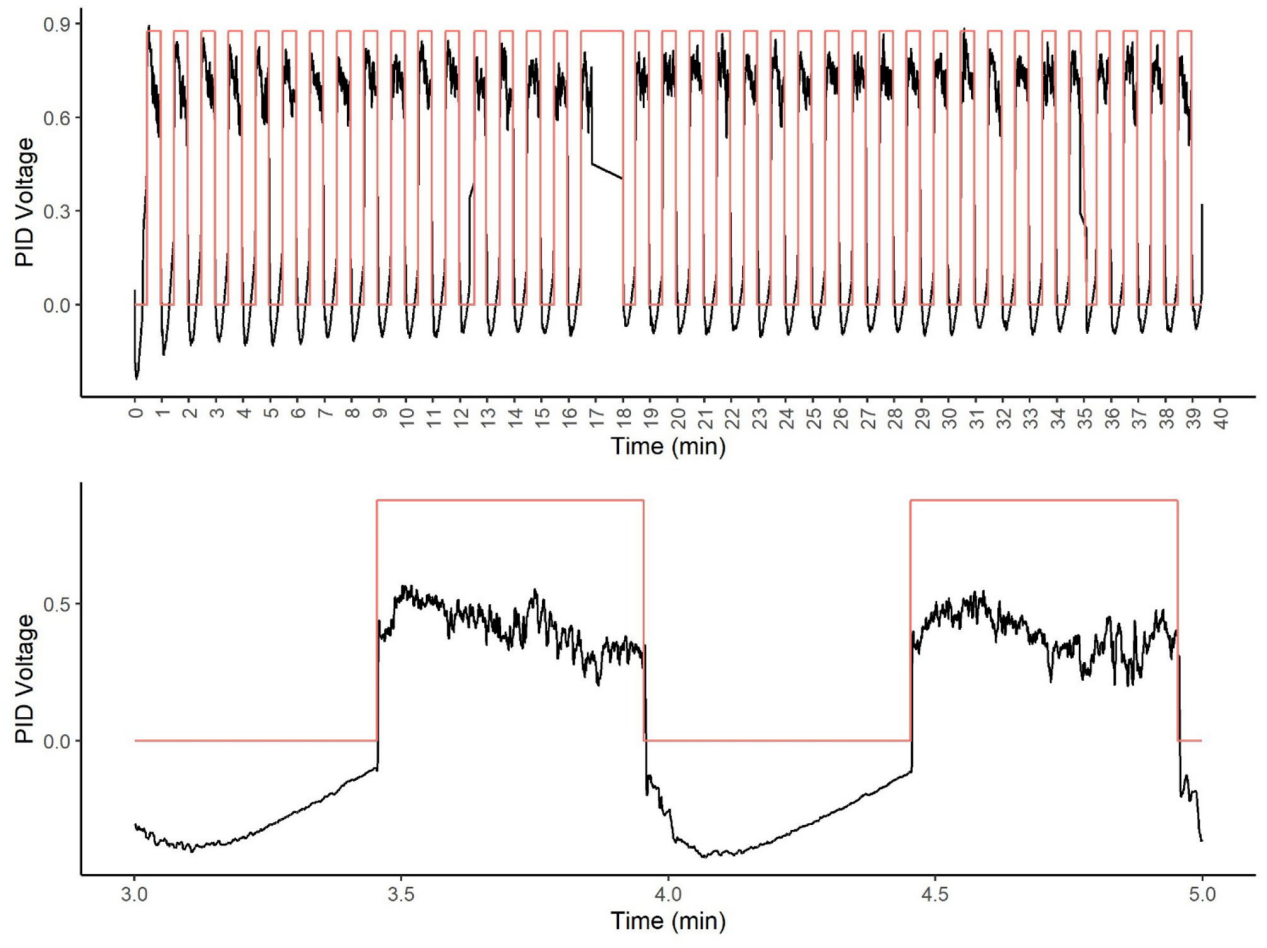
Photoionization detector (PID) analysis of tracer odor. The top figure shows the olfactometer stimulation over a 40-min period. Note at 17 min, a data packet loss from the DAQ occurred. The black line shows the PID voltage. The red line (square wave) shows the olfactometer stimulation (valve on). The bottom figure shows an enlarged view of two representative stimulations.

The PID was able to detect the odor immediately after the activation of the odor valve and was relatively stable over nearly 40 min of stimulation (see Figure 8). Figure 7 shows that odor signal was closely related to olfactometer stimulation (red square wave). Note, one packet of data was dropped from the DAQ between minute 17 and 18. The bottom panel of Figure 7 shows a focused view of two stimulations, highlighting the odor was rapidly detected at the odor port and rapidly cleared from the port with limonene as the tracer odorant.

**Figure 9:**
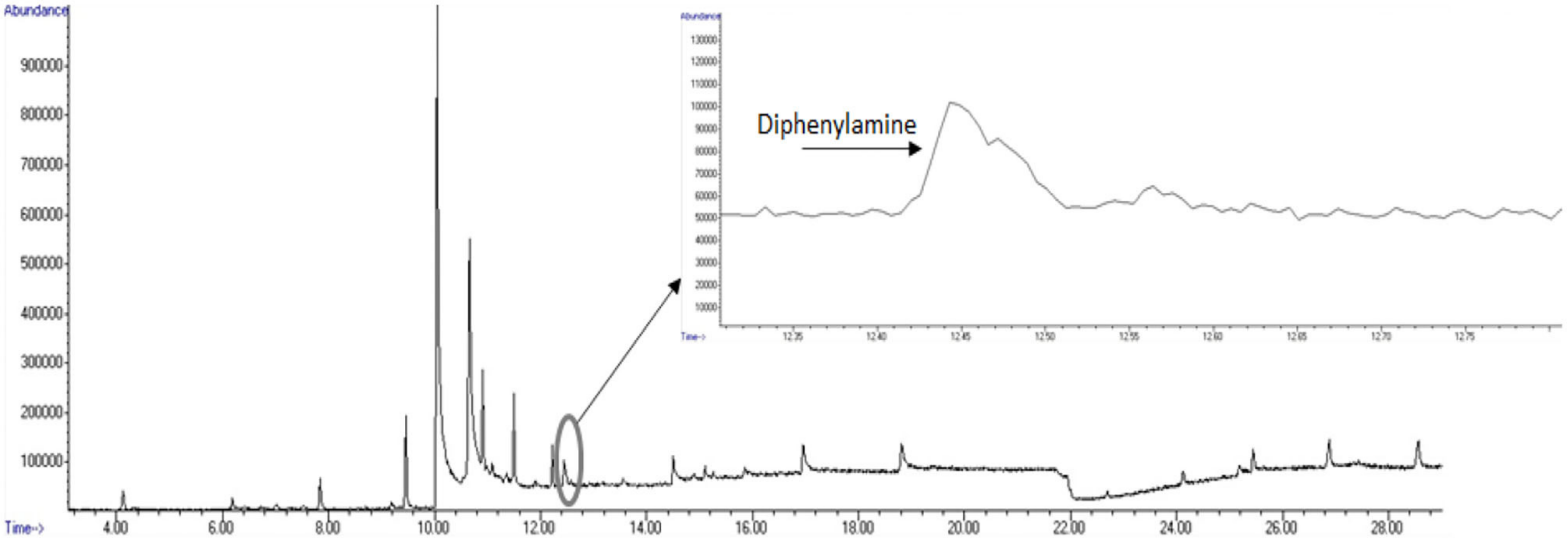
Total ion chromatogram (TIC) of diphenylamine detection from olfactometer sampling.

**Figure 10:**
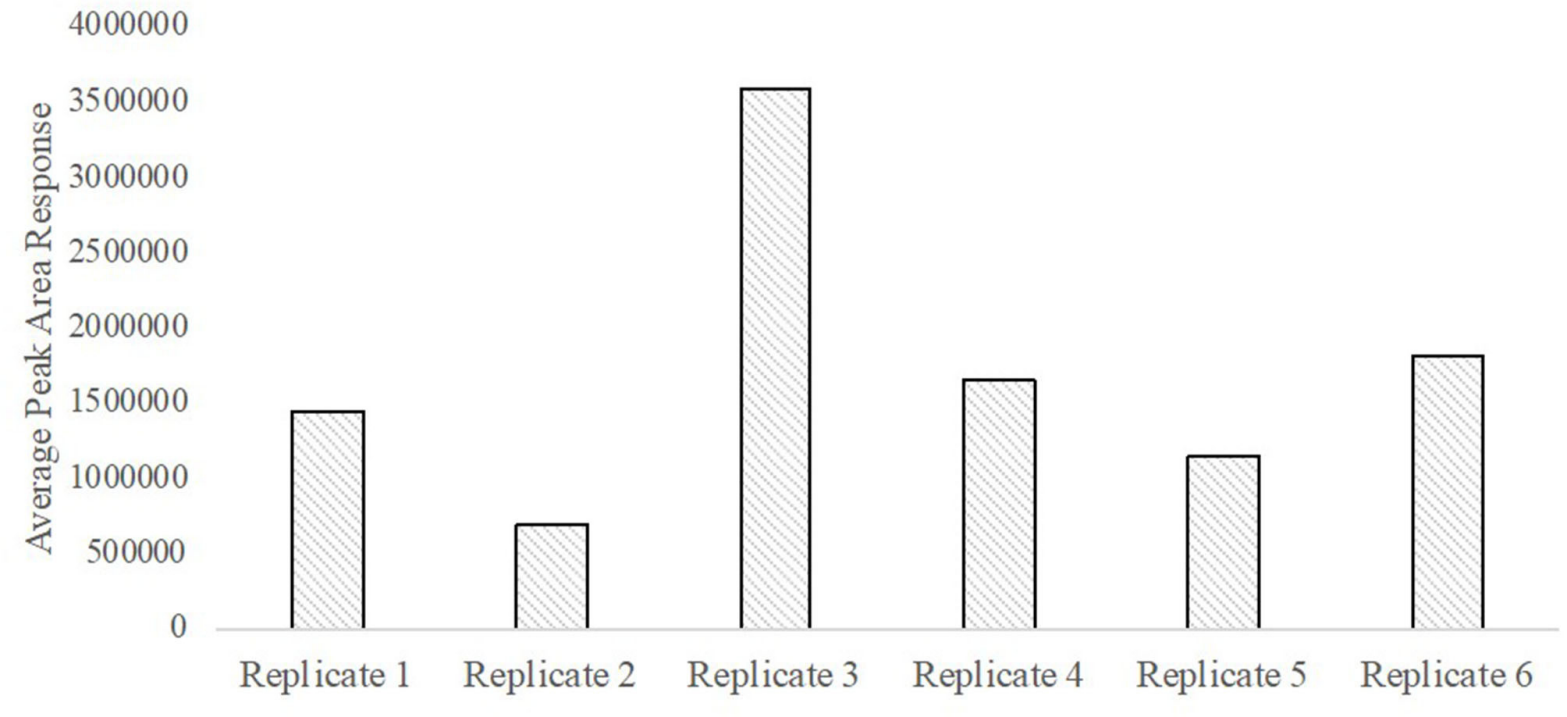
Abundance of diphenylamine obtained from olfactometer output across sampling replicates.

A total ion chromatogram of the SPME-GC/MS analysis is shown in Figure 8 for detection of volatiles associated with the smokeless powder target directly. As seen in Figure 9, the polyacrylate (PA) fiber yielded successful detection of diphenylamine, a target volatile associated with smokeless powder. Diphenylamine was detected in all six of the replicates with an average peak area response of 1,734,916 ± 370,022 (SE) (see Figure 10).

**Figure 11:**
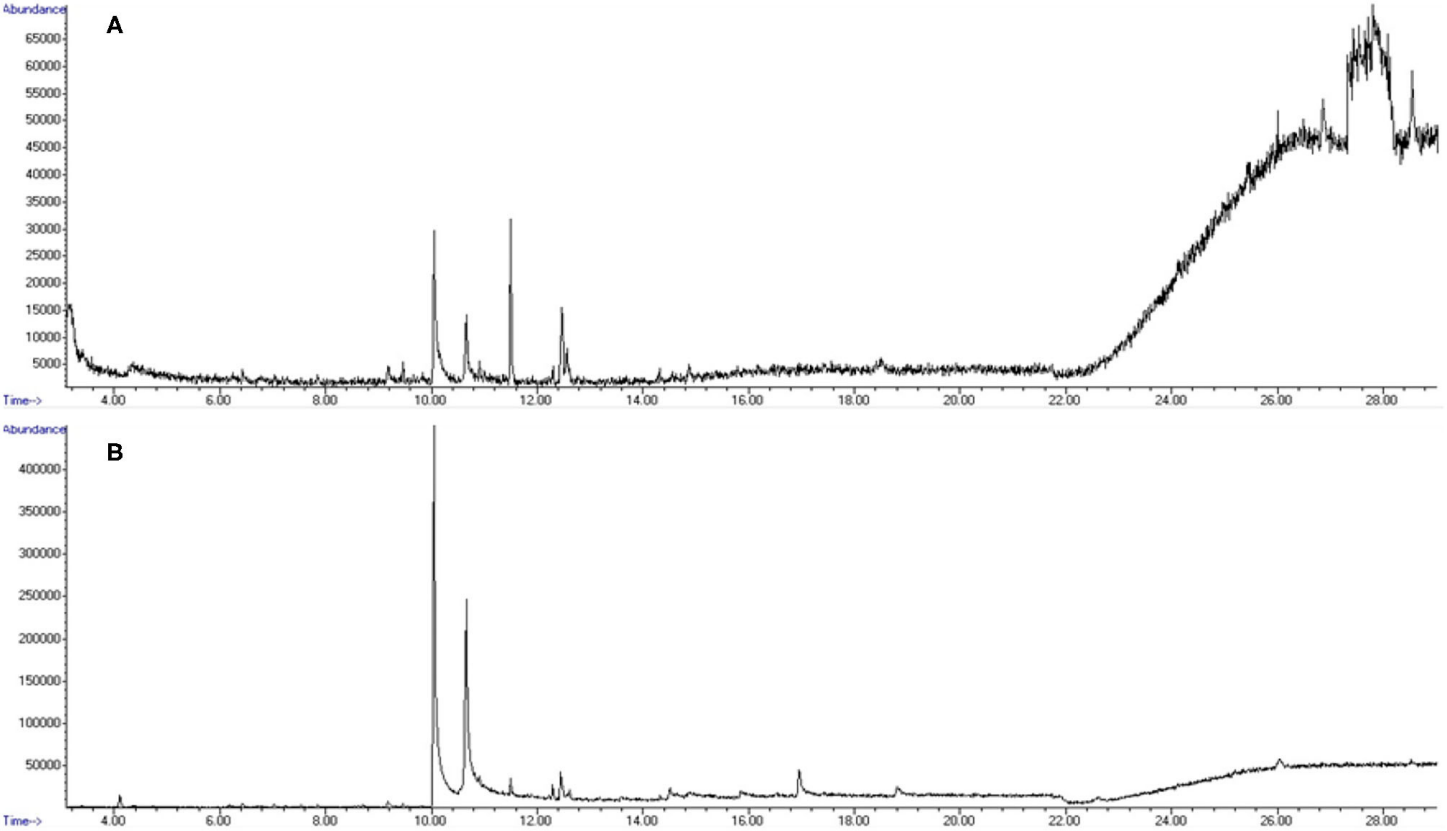
Total ion chromatograms (TIC) from olfactometer blank samplings. **(A)** Pre-powder purge. **(B)** Post-powder purge Note: all shown peaks are attributed to column or environmental background noise.

A total of 18 replicate samples were tested to verify odorant absence from the olfactometer between odor trials. Variations of length of tubing, from 0.30 m (short line) to 0.90 m (long line), were tested, as was the application of heat tape to the shorter line (short line with heat tape) to evaluate if added heat could reduce any potential contaminants present in the system. Six replicates were performed for each variation. Instrumental results did not detect diphenylamine in any of the 18 samples tested. Figure 11 depicts the total ion chromatograms for blank runs extracted before and after odorant purge through the system. Results indicate that only background and column-associated molecules were detected and confirm that there is no detectable carryover or contamination between trials with diphenylamine, suggesting complete removal of the smokeless powder target is achieved within the olfactometer line.

## Discussion

All dogs, presumably naïve to scent detection work, were able to learn and operate the line-up in less than a month. This indicates that the line-up was simple to operate and that dogs can progress through the outlined training method easily. Since we used dogs in a train-for-adoption program, we speculate that working and pet dogs can also learn to operate the apparatus with similar ease. The simplicity of the method is an advantage over other automated systems available. For instance, after screening 12 dogs, only five were trained to perform an automated scent wheel (1). The fact that all 12 participant dogs were able to reach the training criterion is a good indication that most dogs can learn to operate the system.

Traditional scent detection work usually requires extensive training periods. Long training periods are one of the factors that limit the number of subjects that participate in research, as it requires a significant amount of time and resources for each participant to be trained. In a systemic literature review, Johnen et al. (22) found that the average number of participant dogs in detection studies was 4.6 ± 3.2, ranging from one to 10, and that most studies had a training period of over 2 months. The automated line-up system developed could potentially help researchers to increase the number of participant dogs in their studies, as this system requires significantly less training time (less than a month) as it is easy to learn by dogs.

In addition, the automated olfactometer reduces labor. Traditional line-up procedures require researchers to spend a considerable amount of time preparing the sessions ahead of time (e.g., odor preparation, randomization of odor trials, preparing data sheet, etc.) and collecting the data manually during the session or after by coding the recordings. The computer program developed automated all these tasks, significantly reducing the amount of time and effort needed to prepare and run a testing session and to collect the data. In addition, because every trial is generated by the olfactometer program, the system always ensures double-blinded testing. This is another benefit of the apparatus, as it reduces the chances of a dog using unintentional cues from the experimenter (4, 8) (Clever Hans effect). By always ensuring double-blinded conditions, the apparatus increases data reliability as the experimenter can be confident that dogs' performance is not unintentionally biased. For instance, Elliker et al. (23) highlight the importance of double-blind testing in detection dogs as the lack of a robust double-blind testing could lead to erroneous conclusions about dogs' performance. In their experiment, they found that dogs were not able to generalize to new cancer-positive samples during a double-blind testing (23). This suggested that dogs were not alerting to a common cancer odor. The results from our control test show that dogs in the olfactometer were exclusively using odor to make a correct alert, as their performance dropped when the odor vials were not connected to the olfactometer. Because the olfactometer always allows double-blind testing and has incredible odor stimuli control, it is an ideal research instrument to study generalization and/or evaluate if the dogs are solely using a specific odor to alert and confirm that the phenomenon observed by Elliker et al. (23) is not happening. The olfactometer will be a great tool to quickly detect similar problems early in training to prevent researchers from concluding that dogs are alerting to a specific target odor when in reality they are not.

The use of this apparatus significantly increased the amount of data collected from each dog. For instance, on average, each 40-trial session took 30 min or less. In a traditional laboratory line-up model, dogs receive <10 testing trials in a day (22, 24, 25). Johnen et al. (22) reported that only two of the 14 papers reviewed had more than 30 testing replicates, and both studies had a data collection period of over a month. Herein, we collected data for 480 trials per day (e.g., one training session for 12 dogs will take 6–8 h). The capability of increasing the amount of data collection is extremely beneficial to detection dog research as it increases the power of experiments, especially when few participants are used. By significantly increasing the number of trials (“searches”), the apparatus also increases the precision of the variables estimated within a session and/or individual dog.

The apparatus also increased the resolution of the data collected. Using infrared sensors, the apparatus was able to measure not only the amount of port entries but also the exact duration of a nose hold. For instance, the cumulative sniffing time (sum of the amount of time a dog sniffs each port) at the end of training was <1 s. This indicates that a dog sniffed each port for <0.50 s on average. Measuring this would have been extremely difficult by a human observer even when watching video recordings of the sessions. The automated system also provided an objective evaluation of dogs' responses. By using the infrared sensors, the apparatus reduced human error when recording an alert and potential variability between observers (e.g., an observer counts four seconds faster than other) as the 4-s nose hold is always read by the sensor. This provides researchers with an objective and unbiased way to call and record dogs' alerts.

The odor validation data showed how the olfactometer provided an optimal control on the odor stimuli presented. Our results showed that immediately after the valve was activated, the odor was detected in the port. Thus, there was no delay in odor presentation. Similarly, the odor signal returned to baseline immediately after the odor valve was closed. After 40 consecutive 30-s odor on/off cycles, the odor signal remained detectable by the PID. The maximum number of times an odorant could be presented by an olfactometer (the system has three olfactometers) within a session was 13–14 times, indicating that there is good odor stability across a session.

One potential limitation, however, is that in our smokeless powder target, SPME-GC/MS analysis did indicate variability in diphenylamine concentrations across replicates (although it was always present). Peak areas fluctuated across the replicate samplings, suggesting airflow introduction as a possible factor for lack of response reproducibility. Another factor for peak area fluctuation can be related to the dynamic chemistry of smokeless powder, which can affect detection of odor volatiles. Diphenylamine is a stabilizer that may interact with nitrocellulose or nitroglycerin when allowed to stand, thus affecting the overall powder composition at any given moment. The original powder-manufacturing process and environmental conditions determine how each of these is incorporated into powder. These reactions can yield to stabilizers degrading into nitrogenous products not readily detected by the employed methodology (26). However, it should be noted that even with repetitive sampling occurrences, target odor volatile was still detectable for all replicates within instrumental limits.

During training, we conducted each session 5–10 min apart from each other, and we did not notice detection issues reflected in canine performance. However, it is important to note that the odor dynamics are different for each odorant. Factors such as the concentration of the solution, vapor pressure, and the partition coefficient could change the odor dynamics on the olfactometer. Thus, we suggest that an odor validation test should be conducted for each target odor tested.

The apparatus has many benefits to detection dog research, but there are some factors that might limit its utilization in detection dog research. (1) The use of the automated line-up is limited to indoors or laboratory settings. Even when the apparatus could be modified in different ways (e.g., to use battery power, to connect *via* Bluetooth to a laptop or tablet removing the need of wires, to add more ports, to change the position of the ports, etc.), it still is not suitable for testing outdoors as it is sensitive to dust and water. Furthermore, in some instances, the traditional research methods continue being more suitable as they better resemble the actual detection task of working dogs. Thus, at this stage of development, the apparatus is an ideal tool to do basic or proof-of-concept research in a controlled laboratory setting. (2) The cost of the system is another factor that could limit the utilization of the apparatus. The three-port system costs around $5,000 dollars. The cost could be reduced by changing some construction materials, but it is still significantly more expensive than traditional methods such as paint cans. (3) Researchers or trainers must have some minimum level of technical expertise in computer programing to be able to modify the computer program to maximize the use of the equipment and adapt it to different research or training purposes.

## Conclusion

All dogs readily learned to operate the apparatus and search in a line-up manner to alert to a relevant target odorant (smokeless powder). By the end of the training, most dogs achieved overall accuracy levels >90%. All dogs were successfully trained with a 15–23-day window, indicating the apparatus is an efficient tool for training new detection tasks. The device also allowed us to conduct numerous trials per day efficiently while conducting all trials double blind. Control trials further revealed that dogs were utilizing odor cues and not unintentional cues of the system. Instrumental validation verified target odorant detection and confirmed clearance of odor stimuli from device when no odor was in use. Altogether, we conclude that the automated line-up is a useful laboratory equipment for measuring dogs' olfactory performance. The developed automated system maybe a valuable tool to enhance and improve detection dog olfactory research.

## Data Availability Statement

The original contributions presented in the study are included in the article/Supplementary Material, further inquiries can be directed to the corresponding author.

## Ethics Statement

The animal study was reviewed and approved by Texas Tech University Institutional Animal Care and use Committee.

## Author Contributions

NH was the inventor of the apparatus, designed all the schematics, and developed the Arduino and Python algorithms. EA-R built the apparatus and trained the dogs. Data analysis was conducted and the manuscript was written by EA-R and NH. SPME-GC/MS data collection and analysis was conducted by SG. PP-T and SG conducted chemical analyses and participated in experimental design. All authors contributed to the article and approved the submitted version.

## Funding

This research was funded by a contract from the United States Department of Homeland Security (DHS), contract: 70RSAT20CB0000010.

## Author Disclaimer

Any opinions, findings, and conclusion or recommendations expressed in this material are those of the authors and do not necessarily reflect the view of the sponsor.

## Conflict of Interest

The authors declare that the research was conducted in the absence of any commercial or financial relationships that could be construed as a potential conflict of interest.

## Publisher's Note

All claims expressed in this article are solely those of the authors and do not necessarily represent those of their affiliated organizations, or those of the publisher, the editors and the reviewers. Any product that may be evaluated in this article, or claim that may be made by its manufacturer, is not guaranteed or endorsed by the publisher.

## Supplementary Material

Olfactometers Program files for the computer and arduino program are available at: https://github.com/njhall1/CanineOlfactometer
