# Supplementary material for: An Automated Canine Line-Up for Detection Dog Research
Source: Front Vet Sci. 2021 Dec 20;8:775381. doi: 10.3389/fvets.2021.775381 (PMC8771161; doi:10.3389/fvets.2021.775381)
Supplement: Supplementary file 1 [file Table_1.DOCX]

Supplementary Table: Bill of Materials

| Reference# | Supplier | Mfc | Part # | Units/olfactometer | # Olfactometers | Unit/cost | Total Cost/Olfactometer | Description |
| --- | --- | --- | --- | --- | --- | --- | --- | --- |
| 1 | Wilson-Company | Clippard | EV-2M-12-H | 6 | 3 | 27.45 | 988.2 | 2-way manifold mount 12v valve (high flow) |
| 2 | Wilson-Company | Clippard | EV/ET 15481-6 | 1 | 3 | 21.59 | 64.77 | Manifold with 6 mounting ports |
| 3 | Wilson-Company | Dwyer | VFB-65-BV | 1 | 3 | 73.15 | 219.45 | Rotameter 4" scale 0.2-4 Lpm |
| 4 | Wilson-Company | Dwyer | VFB-60-BV | 1 | 3 | 73.15 | 219.45 | Rotameter 4" scale 0-1 Lpm |
| 5 | Industrial Spec | Industrial Spec | [PTFE-11618-50](https://www.industrialspec.com/shop/plastic-tubing/ptfe-fluoropolymer-tubing/ptfe-11618-50.html)  And  [PTFE-1814-50](https://www.industrialspec.com/shop/plastic-tubing/ptfe-fluoropolymer-tubing/ptfe-1814-50.html) | 0.2 | 3 | 317.15 | 190.50 | PTFE Tubing (1/16 x 1/8” and 1/8” x ¼”) |
| 6 | Industrial Spec | Industrial Spec | BHP-2M-SS303 | 1 | 3 | 2.3 | 6.9 | Plug for Clippard manifold |
| 7 | Industrial Spec | Industrial Spec | QCSW-18-1032M-SS316 | 6 | 3 | 9.9 | 178.2 | 10-32 x 1/8" push in fitting for Clippard Manifold |
| 8 | Industrial Spec | Industrial Spec | QCSW-18-2M-SS316 | 6 | 3 | 9.9 | 178.2 | 1/8" NPT x 1/8" push for PTFE manifold fitting |
| 9 | Industrial Spec | Industrial Spec | LNM-6-4F2F-T | 1 | 3 | 72.16 | 216.48 | PTFE manifold |
| 10 | Industrial Spec | Industrial Spec | QCSW-14-4M-SS316 | 2 | 3 | 9.9 | 59.4 | 1/4" NPT x 1/4" push connects tube to PTFE inlet ports |
| 11 | Industrial Spec | Industrial Spec | QCSW-14-2M-SS316 | 4 | 3 | 9.9 | 118.8 | 1/8" NPT x 1/4" Push connect to connect rotameters |
| 12 | Industrial Spec | Industrial Spec | QCSW-14-2M-SS316 | 1 | 3 | 9.9 | 29.7 | 1/8" NPT x 1/4" Push connect to connect for black manifold inlet |
| 13 | Industrial Spec | Industrial Spec | QUCBW-14-SS316 | 1 | 3 | 22.25 | 66.75 | 1/4" push bulkhead (Or 1/8 NPT for odor port) |
| 14 | Industrial Spec | Industrial Spec | QUTW-14-SS316 | 1 | 3 | 31.96 | 95.88 | 1/4" Push T-Junction |
| 15 | Industrial Spec | Industrial Spec | CHH-2F2M-1/3#-V-SS | 6 | 3 | 16.71 | 300.78 | Check valve 1/8" MNPT x 1/8"FNPT 1/3 psi |
|  |  |  |  |  |  |  |  |  |
| 16 | Bowers Plastic | Bowers Plastic | Custom | 1 | 3 | 486 | 1458 | PTFE odor port |
| 17 | Bowers Plastic | Bowers Plastic | Custom | 0.25 | 3 | 286 | 214.5 | black Poly propylene 1/4" |
| 18 | OSH Park | Printed circuit board | Custom | 1 | 3 | 7.66 | 23 | circuit board |
| 19 | amazon | Darlington driver | ULN 2803 | 2 | 3 | 1.5 | 9 | Transistor array |
| 20 | amazon | Arduino nano ble | ArduinoNano BLE 33 | 1 | 3 | 34 | 102 | microcontroller |
| 21 | amazon | 80/20 INC | 10 series T-slot | 1.5 | 3 | 36.48 | 164.16 | Metal channel |
| 22 | amazon | power supply | 12v power | 1 | 3 | 12 | 36 | 12v power |
| 23 | Amazon | Sunon | 12v exhaust fan | 1 | 3 | 13.64 | 40.92 | 40mm x 40mm exhaust fan |
| 24 | Amazon | Adafruit | IR Beam pair  ADA2168 | 1 | 1 | 10.00 | 30 | IR beam pair |
| 25 | Amazon | Many | ¾” NPT Nipple | 1 | 1 | 10 | 30 | Exhaust stainless steel nipple |
| 25 | Home Depot | PVC | PVC fittings | 1 | 1 | 10 | 30 | PVC fitting for exhaust customized to testing area |
|  |  |  |  |  |  |  |  |  |
|  |  |  |  |  |  | **Sum** | **5035.04** |  |
|  |  |  |  |  |  |  |  |  |
|  | **Air System** |  |  |  |  |  |  |  |
| 26 | amazon | air pump | aquarium pump | 0.33 | 3 | 44.99 | 44.5401 | [Link](https://www.amazon.com/VIVOHOME-Electromagnetic-Commercial-Aquarium-Hydroponic/dp/B078H92695/ref=sxts_b2b_sx_reorder?crid=3U0287BZFECNH&cv_ct_cx=aquarium+air+pump&dchild=1&keywords=aquarium+air+pump&pd_rd_i=B078H92695&pd_rd_r=a75640e8-68c6-428a-9c2f-e2e81fcc813a&pd_rd_w=zX13a&pd_rd_wg=UnBkz&pf_rd_p=e3453390-8188-4ed9-a1b3-46cb747551ce&pf_rd_r=KEV13JC6JDAK87J9A736&qid=1594396661&sprefix=aqua%2Caps%2C211&sr=1-1-f5ebfd8e-82c1-4b4e-97d5-2aa47aa18b69) |
| 27 | amazon | air filter | air filter | 0.33 | 3 | 13.72 | 13.5828 | [Link](https://www.amazon.com/Omnipure-K2533JJ-Inline-Filter-Quick-Connect/dp/B00BA9DE94/ref=sr_1_3?dchild=1&keywords=activated+charcoal+water+filter+inline&qid=1594396781&sr=8-3) |
|  |  |  |  |  |  | **Sum** | **58.1229** |  |
|  | **Panel** |  |  |  |  |  |  |  |
| 28 | amazon | Panel Arduino Nano | Arduino Nano | 0.33 | 3 | 34 | 33.66 |  |
| 29 | amazon | 100mm linear rail | motor linear rail | 0.33 | 3 | 69.99 | 69.2901 | [Link](https://www.amazon.com/gp/product/B07DC42DLW/ref=ppx_yo_dt_b_asin_title_o03_s00?ie=UTF8&psc=1) |
| 30 | amazon | Spark fun | Easy driver | 0.33 | 3 | 12.99 | 12.8601 | [Link](https://www.amazon.com/SparkFun-EasyDriver-Stepper-Motor-Driver/dp/B004G4XR60/ref=sr_1_1?dchild=1&keywords=sparkfun+easy+driver+motor&qid=1594576625&sr=8-1) |
| 31 | Bowers Plastic | Bowers Plastic | Custom | 0.04125 | 3 | 286 | 35.3925 | Black Poly propylene 1/4" |
| 32 | amazon | 80/20 INC | 10 series T-slot | 0.495 | 3 | 36.48 | 54.1728 | Metal channel |
|  |  |  |  |  |  | **Sum** | **205.37** |  |
